# Supplementary material for: Bortezomib in Combination with Physachenolide C Reduces the Tumorigenic Properties of KRASmut/P53mut Lung Cancer Cells by Inhibiting c-FLIP
Source: Cancers (Basel). 2024 Feb 4;16(3):670. doi: 10.3390/cancers16030670 (PMC10854725; doi:10.3390/cancers16030670)
Supplement: Supplementary file 1 [file cancers-16-00670-s001.zip › cancers-2759800-supplementary.pdf]

Supplementary Materials: Bortezomib in Combination with Physachenolide C Reduces the Tumorigenic Properties of KRAS^{mut}/P53^{mut} Lung Cancer Cells by Inhibiting c-FLIP

Thanigaivelan Kanagasabai, Zerick Dunbar, Salvador González Ochoa, Tonie Farris, Sivanesan Dhandayuthapani, E. M. Kithsiri Wijeratne, A. A. Leslie Gunatilaka and Anil Shanker

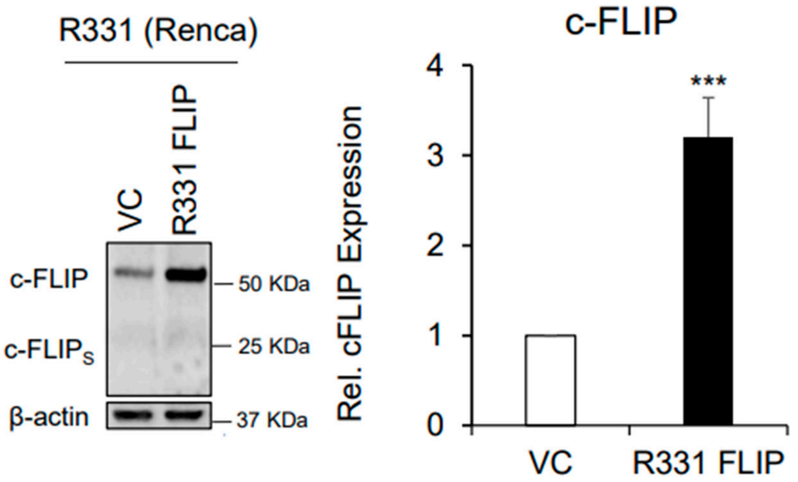

Figure S1. Expression level of c-FLIP in VC and R331 (Renca Clone) overexpressed cell line. Cell lysates from VC (vector control) and FLIP overexpressed R331 cells were prepared followed by Western blotting analysis and quantification analysis of c-FLIP expression was shown. (*** $p < 0.001$).

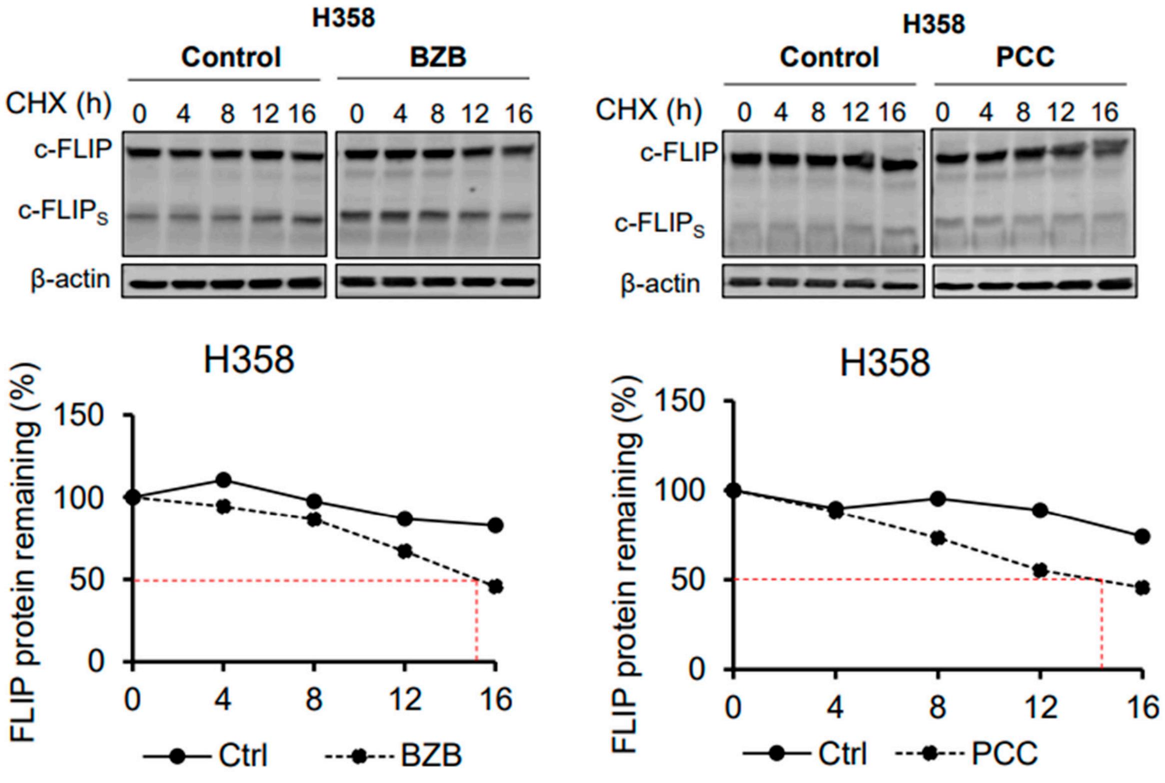

Figure S2. Effects of bortezomib and PCC on c-FLIP protein stabilization. The effects of bortezomib and PCC on c-FLIP protein stabilization was performed using Cycloheximide (CHX) chase assay. Briefly, H358 cells were treated with CHX (100 µg/mL) at indicated time point and cell lysates were prepared followed by Western blotting analysis. Bottom panel: Quantification analysis of c-FLIP protein stabilization. The red dotted line indicates $t_{1/2}$ of the protein.

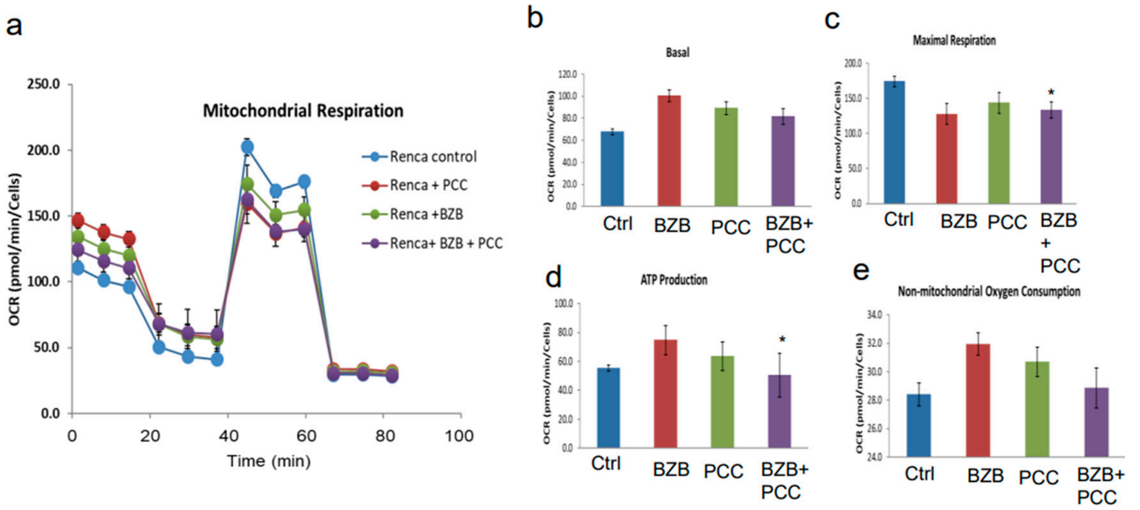

Figure S3. Effects of bortezomib and PCC combination on mitochondrial respiration on renal cancer cells. (**a**) Mitostress assay was performed by assessing oxygen consumption rates (OCR) after 48 h treatment with the bortezomib (20 nM) and PCC (500nM) combination in the presence of oligomycin (3 µM), FCCP (0.25 µM) and rotenone (1 µM) + antimycin (1 µM). (**b**–**e**) Mitostress assay bioenergetic parameters measured from Seahorse results: (**b**) basal respiration, (**c**) maximal respiration and (**d**) ATP production and (**e**) non-mitochondrial oxygen consumption was also assessed as indicated above. OCR values were normalized on protein content measured by SRB assay as per manufacturer's instruction. (* $p < 0.05$).

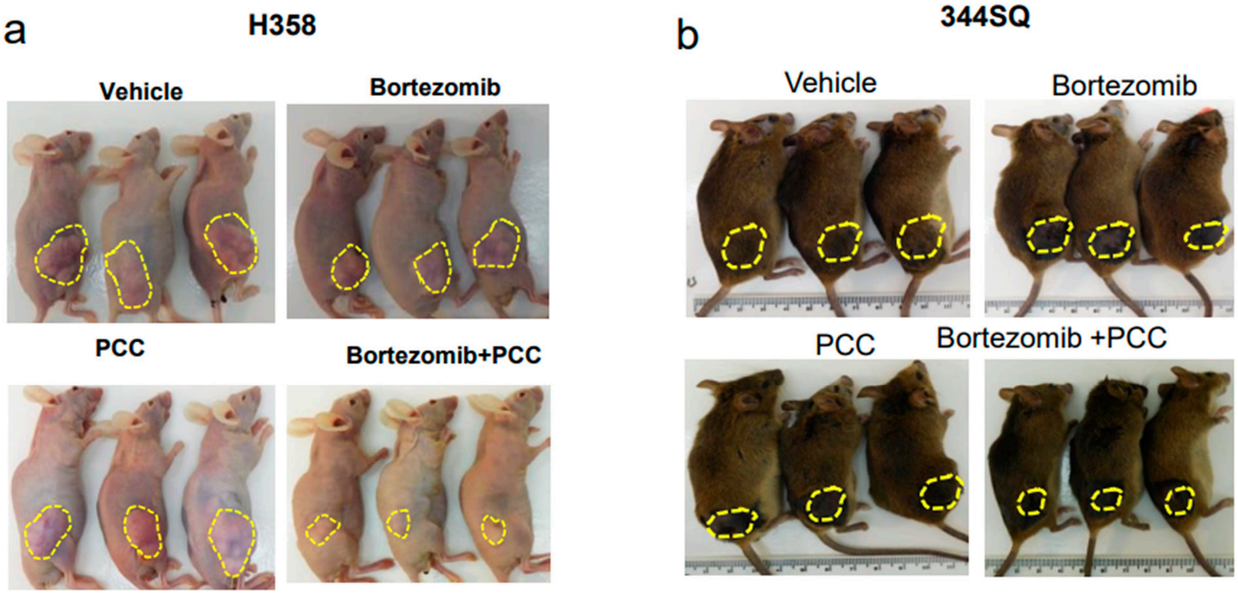

Figure S4. Anti-tumorigenic effects of bortezomib and PCC combination on lung tumor Xenograft mouse model. (**a**) Tumor size reduction in H358, and (**b**) 344SQ lung tumor xenograft mouse models. Tumors are dot-circled in yellow color.

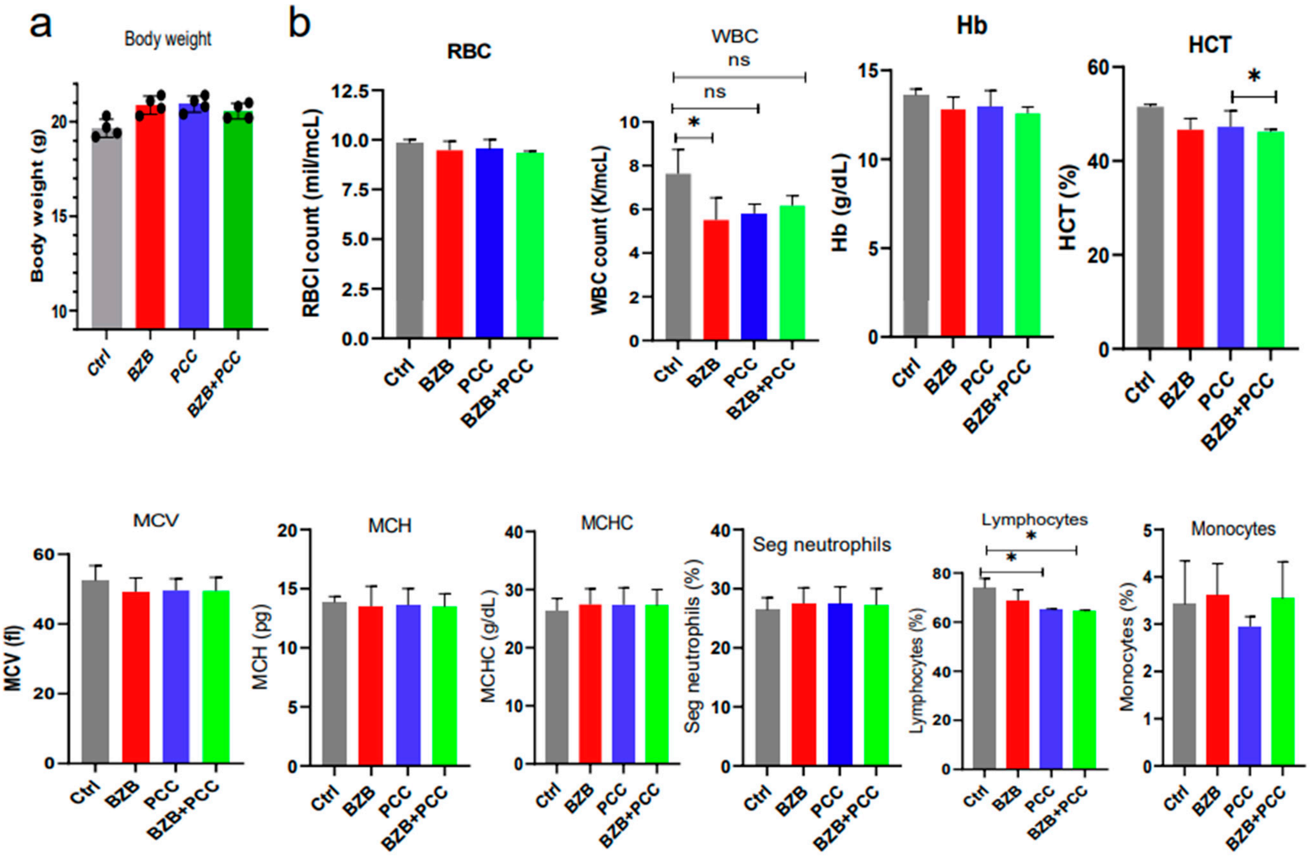

Figure S5. Evaluation of systemic toxicity in mice after bortezomib and PCC treatment. (**a**) Body weight changes in mice, Balb/c mice (3-4/group) were injected with saline (control), PCC (20 mg/kg, s.c.), bortezomib (5 nm, i.p.) or combinaltion (PCC+ BZB) twice a week for 4 weeks. (**b**) Blood parameters including hematological analysis (Red blood cells (RBC), White blood cells (WBC), Hemoglobin (Hb), Hematocrit (HCT), Mean corpuscular volume (MCV), Mean Corpuscular Hemoglobin (MCH), Mean Corpuscular Hemoglobin Concentration (MCHC), Segmented neutrophils, Lymphocytes and monocytes were assessed after the treatment period. (* $p < 0.05$).

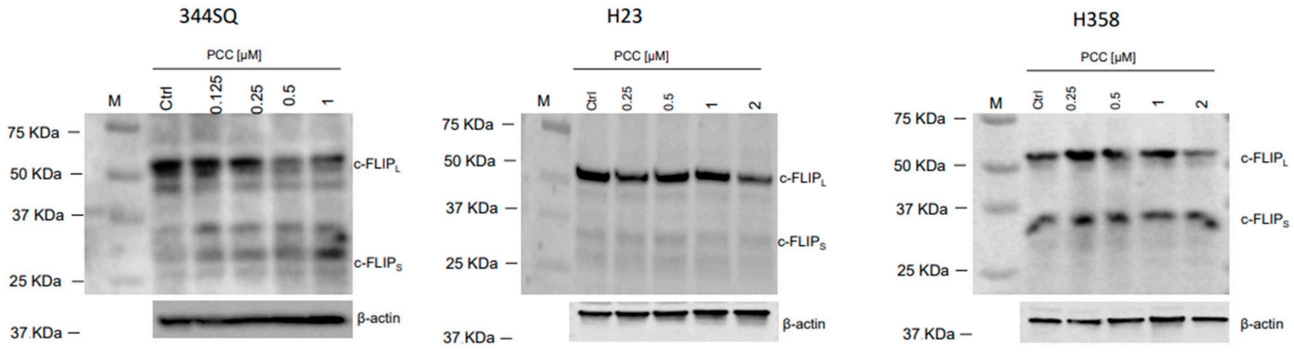

Figure S6. Full Western Blot Images from Figure 1d.

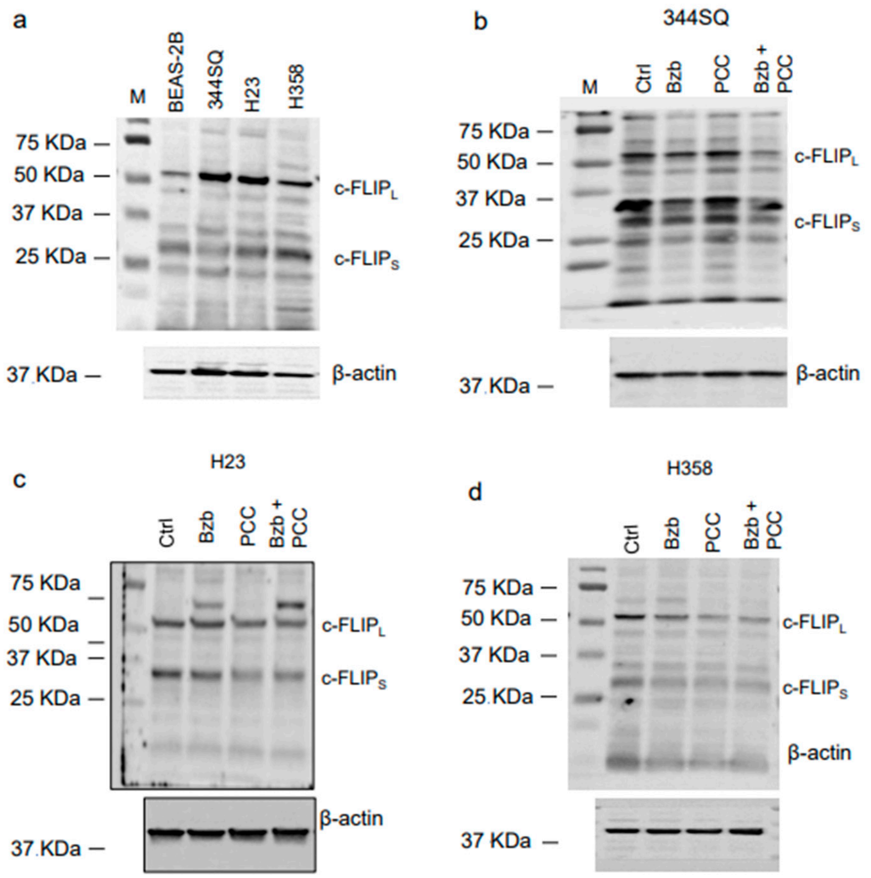

a
M
BEAS-2B
344SQ
H23
H358
75 KDa
50 KDa
37 KDa
25 KDa
c-FLIP$_L$
c-FLIP$_S$
37 KDa
β-actin
b
344SQ
M
Ctrl
Bzb
PCC
Bzb + PCC
75 KDa
50 KDa
37 KDa
25 KDa
c-FLIP$_L$
c-FLIP$_S$
37 KDa
β-actin
c
H23
Ctrl
Bzb
PCC
Bzb + PCC
75 KDa
50 KDa
37 KDa
25 KDa
c-FLIP$_L$
c-FLIP$_S$
β-actin
37 KDa
d
H358
Ctrl
Bzb
PCC
Bzb + PCC
75 KDa
50 KDa
37 KDa
25 KDa
c-FLIP$_L$
c-FLIP$_S$
β-actin
37 KDa

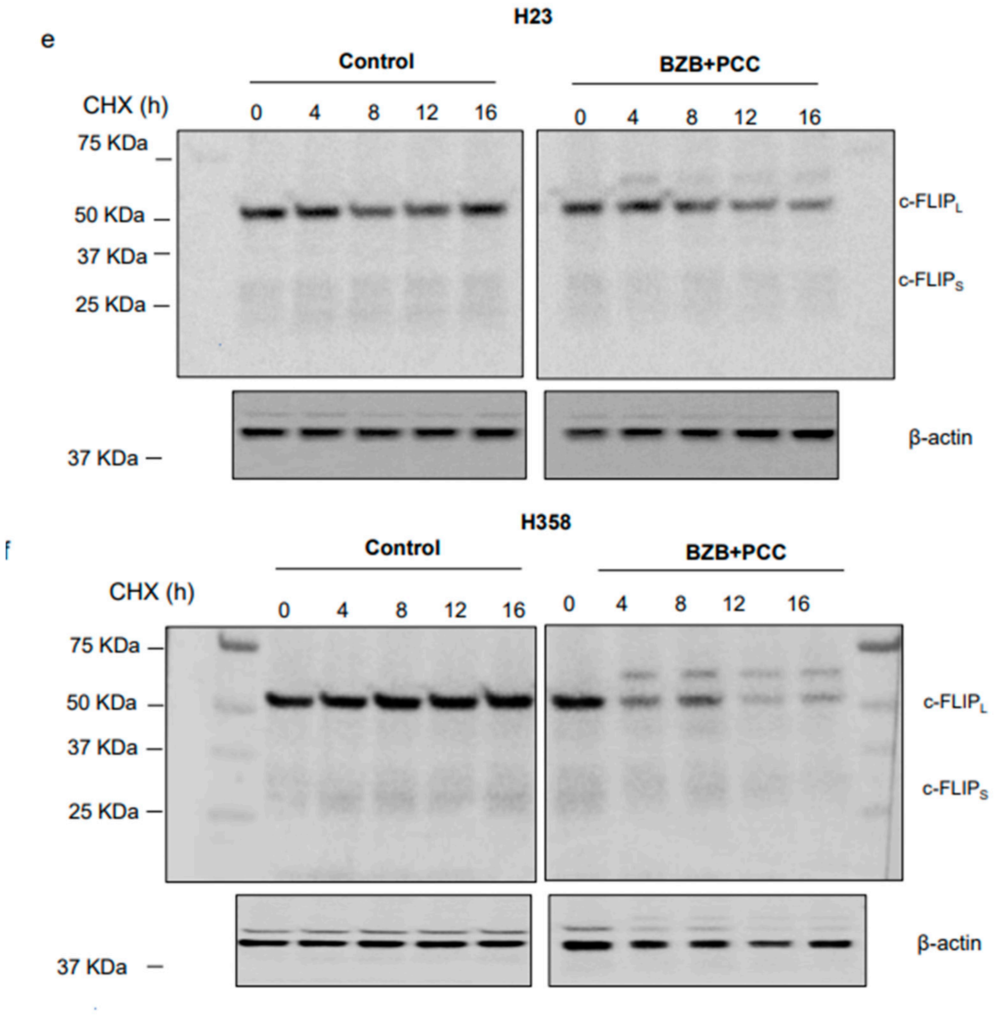

Figure S7. Full Western Blot Images from Figure 3 (**a**–**f**).
